# Increased Salt-Sensitive Blood Pressure in Women vs Men: Is Relative Hyperaldosteronism the Mechanism?

**DOI:** 10.1210/clinem/dgae871

**Published:** 2024-12-19

**Authors:** Ezgi Caliskan Guzelce, Kelly Yin Han Wong, Mahyar Heydarpour, Luminita H Pojoga, Jose Romero, Jonathan S Williams, Gail K Adler, Ellen W Seely, Gordon H Williams

**Affiliations:** Division of Endocrinology, Diabetes and Hypertension, Harvard Medical School, Brigham and Women's Hospital, Boston, MA 02115, USA; Division of Endocrinology, Diabetes and Hypertension, Harvard Medical School, Brigham and Women's Hospital, Boston, MA 02115, USA; Division of Endocrinology, Diabetes and Hypertension, Harvard Medical School, Brigham and Women's Hospital, Boston, MA 02115, USA; Division of Endocrinology, Diabetes and Hypertension, Harvard Medical School, Brigham and Women's Hospital, Boston, MA 02115, USA; Division of Endocrinology, Diabetes and Hypertension, Harvard Medical School, Brigham and Women's Hospital, Boston, MA 02115, USA; Division of Endocrinology, Diabetes and Hypertension, Harvard Medical School, Brigham and Women's Hospital, Boston, MA 02115, USA; Division of Endocrinology, Diabetes and Hypertension, Harvard Medical School, Brigham and Women's Hospital, Boston, MA 02115, USA; Division of Endocrinology, Diabetes and Hypertension, Harvard Medical School, Brigham and Women's Hospital, Boston, MA 02115, USA; Division of Endocrinology, Diabetes and Hypertension, Harvard Medical School, Brigham and Women's Hospital, Boston, MA 02115, USA

**Keywords:** sex differences, salt-sensitive blood pressure, aldosterone, plasma renin activity

## Abstract

**Context:**

Women vs men have more salt-sensitive blood pressure (SSBP) and higher stimulated aldosterone (ALDO) levels, suggesting that their increased SSBP is secondary to a relative hyper-ALDO state. Contrariwise, men vs women have higher sedentary ALDO levels.

**Objective:**

The present project was designed to address the question are women vs men in a relatively hyper-ALDO state.

**Methods:**

A total of 363 women and 483 men were selected from the HyperPATH cohort to assess the potential underlying mechanism for observed sex differences.

**Results:**

Women had greater SSBP, greater ALDO and vasculature response to angiotensin II (Ang II), and higher upright ALDO/ plasma renin activity, but men on both restricted- and liberal-salt diets had higher basal levels of supine ALDO, PRA levels, and other ALDO secretagogues. Using 24-hour urine ALDOs to assess overall production, ALDO did not differ by sex regardless of salt intake, except when assessed in subsets. Normotensive women vs men had greater urine ALDO, and women vs men younger than 51 had higher urine ALDO.

**Conclusion:**

1) Lower Ang II responsiveness in Ang II–targeted organs was observed in men vs women. 2) Similar 24-hour urine ALDO levels in women and men do not support the concept that relative hyper-ALDO is the mechanism for sex difference in SSBP. The data also suggest that the SSBP in women, in some cases, may be benign since it is secondary to a BP reduction on the restricted-salt diet, not an increase on the liberal-salt diet.

Hypertension (HTN) is one of the leading causes of death worldwide and the primary risk for strokes and coronary artery disease. Salt-sensitive blood pressure (SSBP) is common in HTN and has been reported in approximately half of all hypertensive individuals (1). This phenotype also is associated with worse cardiovascular outcomes compared to HTN per se. Of the several factors that have been proposed to underlie the SSBP, the most frequently mentioned is an alteration in the renin-angiotensin (Ang II)–aldosterone (ALDO) system (RAAS).

Several reports have documented sex differences in ALDO and renin with varying results (2-4). However, we could not identify any report that compares both ALDO and renin levels between sexes when age, disease state, and sodium intake are controlled. One publication reported that ALDO was significantly higher in men than in women during a liberal-salt diet but during a restricted-salt diet, this difference was no longer statistically significant. Furthermore, they found significantly lower active plasma renin concentration in men than in women, irrespective of salt intake (5). This was in contrast to earlier studies (3, 4), which describe a lower plasma renin in premenopausal women than in men.

Our group has reported previously that the change in systolic blood pressure (SBP) from a liberal- to a restricted-salt diet (magnitude of SSBP) is 30% higher in women than men, regardless of age and HTN status (6). Recent reviews reported the same results (7). We have suggested that the increased risk of SSBP in women is secondary to a greater ALDO response to infused Ang II, regardless of the level of salt intake. Thus, the greater sensitivity of the adrenal glomerulosa cell to a major secretagogue—Ang II—results in a relatively hyper-ALDO state, increased Na^+^ retention, and SSBP. However, this previous study did not address the finding that on a liberal-salt diet, men had higher supine serum ALDO levels, thereby raising the possibility that 1) a hyper-ALDO state was not more common in women than men and, therefore, 2) not the cause of the increased SSBP in women. The present study is designed to resolve these questions and therefore determine whether women or men are more susceptible to ALDO-mediated adverse conditions.

## Materials and Methods

### Sex as a Biological Variable

The study design accounted for sex as a biological variable. In this observational study, men and women (846 participants) were selected from the International Hypertensive Pathotypes Cohort (HyperPATH) study; 363 were women, and 483 were men. Individuals were recruited from the general populations of Boston, Massachusetts; Salt Lake City, Utah; Rome, Italy; and Paris, France. HyperPATH is a 30-year ongoing program that studies the genetic underpinnings of the hormonal mechanism of HTN and cardiovascular disease (8-11) (supplementary material (12)). To test our hypothesis, we restricted our study cohort to the HyperPATH participants who had measurements of each of our 4 main outcome variables (ALDO on a restricted and liberal-salt diet, plasma renin activity [PRA] on a restricted and liberal-salt diet). We excluded all individuals who were diagnosed with diabetes and also excluded races other than Black and White individuals. Thus, the study cohort included normotensive and hypertensive individuals, White and Black individuals; and participants in 2 age classes (age ≤51 years and >51 years). Although results from the HyperPATH cohort have been previously reported many times, the present data have been reported only in part previously. Specifically, the data comprising the new subcohort have not been described before.

### Statistical Analysis

We used an unpaired *t* test with log transformation for nonnormally distributed variables. Binary variables were compared using the Fisher exact test. Continuous variables are presented as least square means ± SEM. We used a mixed-model, repeated-measures analysis to evaluate the primary end point of assessing the effect of sex differences on baseline supine ALDO, PRA, and Ang II–stimulated ALDO and PRA. In these analyses, nonnormally distributed variables were log-transformed, for example, ALDO, PRA, and cortisol. Covariates (fixed effects) were chosen for their clinical importance (site, disease state, race, age, sex, and body mass index [BMI]) and a random intercept and participant ID were used for the mixed effect. Two-sided *P* values of less than .05 were considered statistically significant. All statistical analysis was performed using JMP 16.

### Study Approval

The institutional review board of each institution approved the original study protocol. All participants gave written informed consent before enrollment and underwent identical protocols at each study site.

## Results

### Participant Characteristics

Age, BMI, race, and disease status distribution were similar between women and men in the study cohort. There was a statistically significant sex difference between Black and White participants, with 9.1% of Black individuals being female and 33.8% of White individuals being female (Table 1).

**Table 1.** Participant characteristics

Sex	Women	Men	
No. (%)	363 (42.9%)	483 (57.1%)	
Variable			*P*
Race, N (%)			
White	286 (33.8%)	424 (50.1%)	.0006
Black	77 (9.1%)	59 (7%)	
Disease, N (%)			.9
Normotensive	128 (15.1%)	169 (20%)	
Hypertensive	235 (27.8%)	314 (37.1%)	
Age (SEM)	45.5 (0.6)	44.5 (0.5)	.2
BMI (SEM)	27.5 (4.8)	27.2 (4.2)	.3

Binary variables: race, and disease state were compared using the Fisher exact test are shown as number and percentages. Continuous variables age and BMI are shown as mean and SEM in parenthesis.

Abbreviation: BMI, body mass index.

### Supine, Unstimulated Aldosterone Levels Are Significantly Higher in Men Than in Women

**Figure 1.**
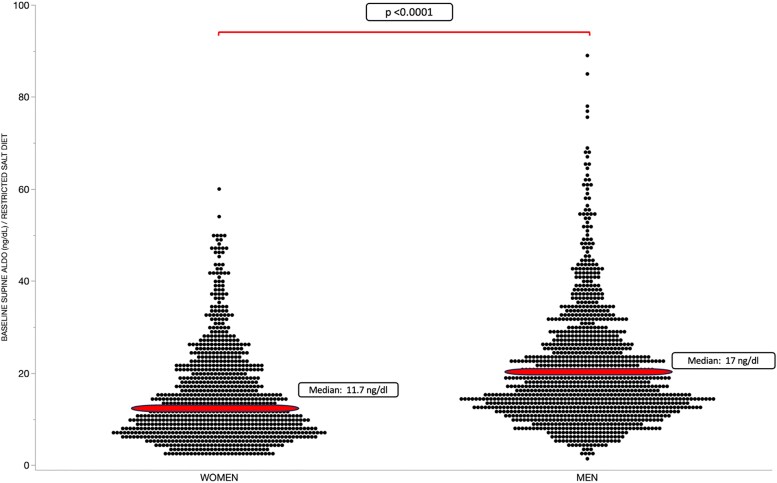
Baseline supine aldosterone (ALDO) (ng/dL) levels are on a restricted-salt diet. Each dot represents the individual's aldosterone measurement. Horizontal lines represent the median. Men (n = 483) had higher baseline supine ALDO levels on a restricted-salt diet than women (n = 363).

**Figure 2.**
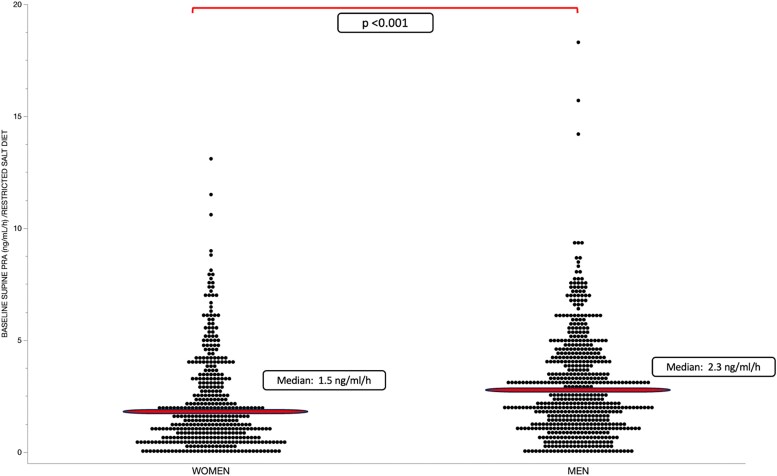
Baseline supine aldosterone (ALDO) (ng/dL) levels are on a liberal-salt diet. Each dot represents the individual's aldosterone measurement. Horizontal lines represent the median. Men (n = 483) had higher baseline supine ALDO levels on a liberal-salt diet than women (n = 363).

In the mixed-model, repeated-measures analysis, baseline supine ALDO levels were statistically significantly (*P* < .0001) higher in men than women both on a restricted-salt and a liberal-salt diet (Figs. 1 and 2).

### Aldosterone's Principal Secretagogue Levels Are Significantly Higher in Men Than in Women

**Figure 3.**
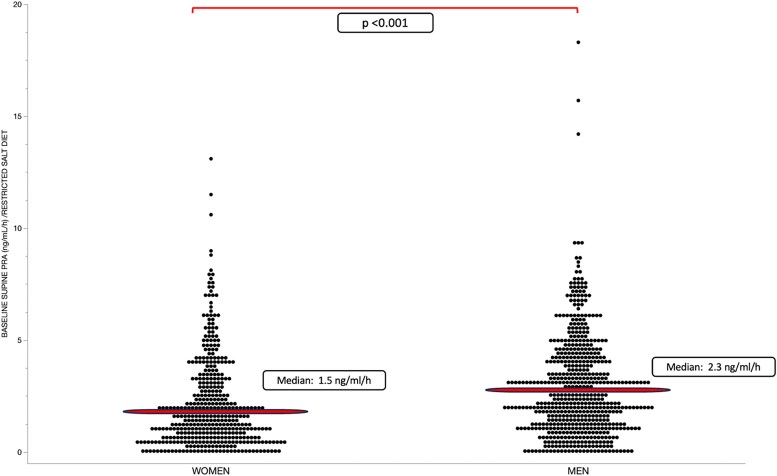
Supine plasma renin activity (PRA) levels on a restricted-salt diet. Each dot represents the individual's PRA measurement. Horizontal lines represent the median. Men (n = 483) had higher baseline supine PRA levels on a restricted-salt diet than women (n = 363).

**Figure 4.**
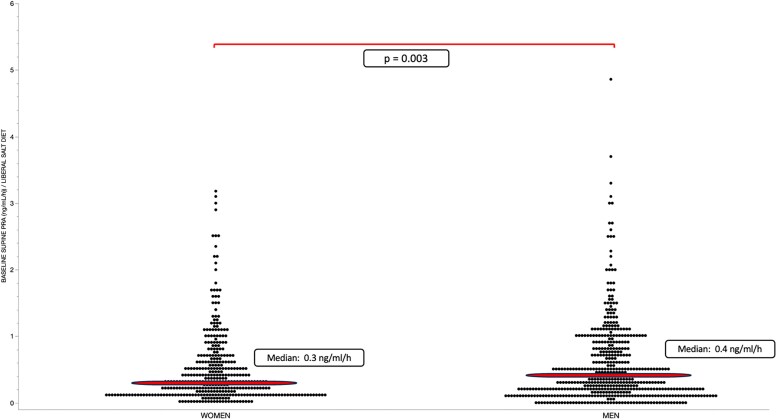
Supine plasma renin activity (PRA) levels on a liberal-salt diet. Each dot represents the individual's PRA measurement. Horizontal lines represent the median. Men (n = 483) had higher baseline supine PRA levels on a liberal-salt diet than women (n = 363).

Renin, via its action on Ang II formation, potassium (K^+^), and adrenocorticotropin (ACTH) are major ALDO secretagogues. Baseline supine PRA levels were higher (*P* < .0001) in men than in women both on a restricted-salt and a liberal-salt diet, similar to the baseline ALDO levels (Figs. 3 and 4).

Endogenous Ang II levels also were higher in men than women on a restricted-salt diet (*P* = .01) in a subset of participants (n = 530) (Supplementary Table S2) (12). This result is consistent with the higher PRA levels and concomitant higher ALDO levels in men than in women at baseline on a restricted-salt diet.

Serum K^+^ values were in the normal range; however, serum K^+^ levels were higher in men than in women on both restricted- and liberal-salt diets (*P* = .01) (Supplementary Table S1) (12). Because the glomerulosa cells are as sensitive to K^+^ as to Ang II (13), the greater levels of these secretagogues in men than in women could contribute to the higher ALDO levels. However, higher K^+^ levels would tend to reduce PRA, and therefore, the differences in K^+^ levels are not likely to contribute to the higher PRA levels.

Serum and urine cortisol levels were statistically higher in men than women on restricted (*P* = .01; *P* <.0001) and liberal (*P* = .007; *P *<.0001) salt diets (Supplementary Table S1) (12). Assuming that cortisol is a reliably reflecting ACTH levels, our results confirm previous reports on serum and urine cortisol (11, 14-16).

Thus, under supine, basal conditions, men as compared with women have increased levels/activity of the 3 major aldosterone secretagogues (K^+^, renin, and ACTH) and increased levels of serum ALDO levels.

### Aldosterone Responses to Angiotensin II Stimulation Are Significantly Higher in Women Than in Men

Women had statistically significantly (*P* < .0001) higher responses to exogenous Ang II stimulation than men on restricted- and liberal-salt diets (Table 2). Further, women had significantly higher absolute and delta ALDO levels in response to upright posture than men (*P* = .008; *P* < .0001, respectively) (Table 3). In contrast, in response to the upright posture, men had higher PRAs than women despite their lower ALDO response (see Table 3).

**Table 2.** Angiotensin II–stimulated supine aldosterone levels in ng/dL

	Women	Men	*P*
Restricted-salt diet			
Ang II–stimulated ALDO, ng/dL (N **=** 828)	35.9 ± 1.2	30.3 ± 1.4	.006
Delta ALDO (Ang II stimulated ALDO – supine ALDO), ng/dL (N **=** 828)	21.4 ± 1.4	12.9 ± 1.5	<.0001
Liberal-salt diet			
Ang II–stimulated ALDO, ng/dL (N **=** 655)	12.7 ± 0.7	11.4 ± 0.7	.09
Delta ALDO (Ang II–stimulated ALDO – supine ALDO), ng/dL (N **=** 655)	8 ± 0.6	6.2 ± 0.6	.03

Data are shown as least square mean ± SEM. Women had greater absolute ALDO response to Ang II than men on the restricted-salt diet, and delta ALDO response to Ang II than men on both diets.

Abbreviations: ALDO, aldosterone; Ang II, angiotensin II.

**Table 3.** Upright posture aldosterone and plasma renin activity levels on a restricted-salt diet

	Women	Men	*P*
Restricted-salt diet			
Upright ALDO, ng/dL (N **=** 803)	43.9 ± 2.2	37.2 ± 2.5	.008
Upright PRA, ng/mL/h (N **=** 804)	6 ± 0.4	7.8 ± 0.5	<.0001
Upright ALDO, ng/dL/Upright PRA, ng/mL/h (N **=** 803)	27 ± 3.3	21.4 ± 3.7	<.0001
Delta upright ALDO (upright ALDO – supine ALDO), ng/dL (N **=** 711)	30 ± 2	19.2 ± 2.3	<.0001
Delta upright PRA (upright PRA – supine PRA) (N **=** 804)	4 ± 0.4	4.9 ± 0.5	<.0001
Delta upright ALDO, ng/dL/Delta upright PRA, ng/mL/h (N **=** 774)	13.6 ± 2.3	8.2 ± 1.2	<.0001

Data are shown as least square mean ± SEM. Women had greater absolute and delta ALDO response to upright position than men, despite men having greater absolute and delta PRA than women. Absolute and delta ALDO/PRA ratios were higher in women than men.

Abbreviations: ALDO, aldosterone; PRA, plasma renin activity.

### Vasculature Response to Angiotensin II Is Significantly Higher in Women Than in Men

To determine the generalizability of the ALDO response to Ang II, we analyzed renal plasma flow and SBP responses to Ang II. Baseline supine and Ang II–stimulated renal plasma flow measured by para-aminohippuric acid clearance both on a restricted- and a liberal-salt diet were similar between women and men. However, delta renal plasma flow response to Ang II was greater in women than men (*P* < .0001) (Supplementary Table S3A) (12). Similarly, while baseline and Ang II–stimulated SBP did not differ by sex, delta SBP in response to Ang II was greater in women than men both on restricted- and liberal-salt diets (*P* = .009) (Supplementary Table S3B) (12). Thus, in women the ALDO, renal plasma flow, and SBP responses to exogenous Ang II were greater than in men.

### Men vs Women Had Higher Baseline Serum Aldosterone and Plasma Renin Activity Levels Regardless of Disease State and Race, but in Men and Women, Younger vs Older Individuals Had Higher Aldosterone and Plasma Renin Activity Levels

In addition to the overall effect of biologic sex on ALDO and PRA levels noted earlier, within each sex group, in both sexes there was an age effect. Women and men aged 51 years and younger had higher baseline supine ALDO (*P* < .0001) and PRA (*P* = .0002) than men and women older than 51 years. We also analyzed 2 other potential factors that might be mediating the different sex-mediated ALDO and PRA responses, namely, disease state (hypertensive/normotensive) and race status (Black or White race). In all conditions regardless of age, men had statistically significantly higher ALDO levels than women, specifically on a restricted diet (Supplementary Table S4) (12).

### Women Have Greater Salt-Sensitive Blood Pressure Than Men Due to Lower Blood Pressure on a Restricted-Salt Diet

**Figure 5.**
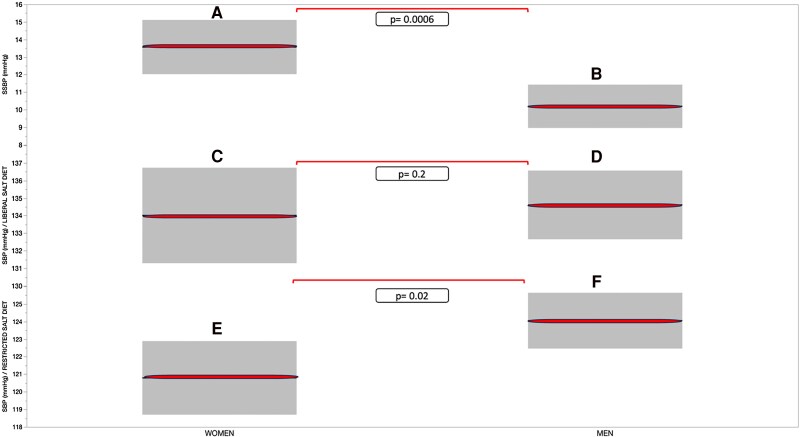
Middle line shows the mean, and the box represents the SD for each variable. (A) Mean SSBP (mm Hg) in women (n = 342). (B) Mean SSBP (mmHg) in men (n = 425). (C) Mean SBP (mm Hg) on a liberal-salt diet in women (n = 349). (D) Mean SBP (mm Hg) on a liberal-salt diet in men (n = 442). (E) Mean SBP (mm Hg) on a restricted-salt diet in women (n = 346). (F) Mean SBP (mm Hg) on a restricted-salt diet in men (n = 437).

As we reported previously, sex differences in SSBP are present (Fig. 5). The average SSBP level was higher in women than men. However, this was not secondary to a greater percentage of women vs men having SSBP as a category variable. The percentage of individuals with SSBP was similar in men vs women irrespective of whether SSBP was defined as a level greater than 7, 10, or 14 mm Hg. Further, women had higher SSBP than men, primarily due to lower SBPs on the restricted-salt diet rather than increased BP on a liberal-salt diet (see Fig. 5 and Supplementary Table S3B) (12). This was potentially secondary to their lower PRA-Ang II-ALDO response to the chronic stress/stimulus of a restricted-salt diet.

There was a statistically significant, but modest, correlation between ALDO levels, ARR on a liberal-salt diet, and SSBP that was driven primarily by their relationships in women vs men (Table 4).

**Table 4.** Correlations of salt-sensitive blood pressure and aldosterone on a liberal-salt diet, and baseline, supine aldosterone/plasma renin activity in the whole cohort, women, and men

Correlation with SSBP	ALDO on a liberal-salt diet	ARR on a liberal-salt diet
Whole cohort (N = 819)	*P* = .038; *r* = 0.08	*P* < .0001; *r* = 0.2
Women (N = 355)	*P* = .006; *r* = 0.14	*P* = .002; *r* = 0.17
Men (N = 464)	*P* = .5	*P* = .0005; *r* = 0.17

*r* represents the correlation coefficient.

Abbreviation: ALDO, aldosterone; ARR, aldosterone/plasma renin activity; SSBP, salt-sensitive blood pressure.

### Twenty-Four–Hour Aldosterone Measurements Are Not Significantly Higher in Women Than in Men

Because women had higher stimulated ALDO levels and men had higher basal ALDO levels, we assess whether a 24-hour ALDO would support a hyper-ALDO state in women vs men in our participants. The 24-hour urine levels were similar between men and women on both a restricted-salt and a liberal-salt diet (Table 5). However, when stratified into subset groups, normotensive women had greater 24-hour urine ALDO levels than normotensive men (*P* = .02), and women younger than 51 had higher 24-hour urine ALDO than men younger than 51 (*P* = .04) (see Supplementary Table S4) (12).

**Table 5.** Supine aldosterone in ng/dL, 24-hour urine aldosterone, and plasma renin activity levels on a restricted- and liberal-salt diet

	Women	Men	*P*
Restricted-salt diet			
24-hour urine ALDO (N **=** 671)	10.7 ± 0.6	11.4 ± 0.6	.3
24-hour urine sodium (N **=** 721)	16.7 ± 1	17.7 ± 1	.4
Liberal-salt diet			
24-hour urine ALDO, ng/dL (N **=** 664)	37.7 ± 1.9	35 ± 2.1	.2
24-hour urine sodium (N **=** 815)	197.8 ± 4.7	210.7 ± 5.1	.02

Data are shown as least square mean ± SEM. On a restricted-salt diet, women and men had similar 24-hour urine ALDO and sodium levels. However, despite having similar 24-hour urine ALDO on a liberal-salt diet, men had higher 24-hour urine sodium than women.

Abbreviation: ALDO, aldosterone.

## Discussion

This study agrees with our previous report (6) in the following characteristics: in women, greater ALDO response to Ang II and higher levels of SSBP; and in men greater basal levels of ALDO on a liberal-salt intake. The present study expands on these findings, allowing for a better understanding of the regulation of ALDO in men vs women. Thus, in the basal, supine state, independent of salt intake, men vs women have significantly higher measurements of ALDO and its 3 major secretagogues, PRA and Ang II, potassium, and cortisol (a surrogate for ACTH). In contrast in women vs men, with administration of Ang II, independent of salt intake or upright posture, not only is ALDO greater but in addition the vascular responses to Ang II, increases in BP and renal plasma flow, are greater in women vs men. Because of these biologic sex-associated differences in serum ALDO levels, we then assessed 24-hour urine ALDO levels as an integrator of an overall ALDO state. There were no differences. Thus, one would assume that neither men nor women are in a hyper-ALDO state. However, 3 caveats temper that conclusion. First, women younger than 51 years have greater 24-hour urine ALDO levels compared to men. Second, normotensive women compared to men have greater 24-hour urine ALDO levels. Third, women, in general, have lower PRA levels.

### Why Are Basal Aldosterone Levels Higher in Men Than in Women?

Circulating ALDO levels, for the most part, are a product of its production and metabolism rates. Its production rate usually is determined by intrinsic zona glomerulosa properties and its secretagogue levels with Ang II, potassium and ACTH being the most prominent ones. Ang II and potassium appear to be equally potent and sustainable, with endogenous ACTH having a variable effect. Since all 3 secretagogues are increased in men vs women basally, to a variable extent each likely contributes to this sex effect. This conclusion suggests that the “set point” between at least Ang II and K^+^ in their classical negative feedback loops differs between men and women (lower in women) with several physiologic and pathophysiologic sex-based consequences. Support for this hypothesis comes from 3 observations. First, women have more SSBP than men in the presence of lower basal ALDO levels. Second, this increases the likelihood that a non-ALDO mechanism is causing the increased SSBP in women. The most likely mechanism would be a reduced renal plasma flow response to Ang II on the liberal-salt diet. The opposite occurred in women vs men, providing support for an altered ALDO response to salt-loading. Third, ALDO/PRA levels were higher in women vs men, again supportive of an enhanced glomerulosa cell response to its secretagogue.

Alternatively, the answer is in the secretagogues, not the glomerulosa cell. If we assume that women have increased SSBP via a non-ALDO mechanism, this will be accompanied by volume expansion resulting in suppressed PRA and also decreased serum potassium in women vs men. However, this enhanced relationship between ALDO and vascular tissues responses to Ang II in women vs men was present regardless of the level of salt intake. At a population level, this enhanced relationship could be modified by genetic, race, age, or environmental factors. Several of these factors were present in the HyperPATH database. In the ones available, the relationship between ALDO, PRA, and SSBP and biological sex remained the same.

### Why Are Aldosterone Responses to Angiotensin II Greater in Women Than in Men?

It is important to point out that the enhanced responses to Ang II were not limited to the adrenal glomerulosa cells but also to BP and renal plasma flow, strongly supporting the concept that they are related to differences in the status of the angiotensin receptor. The responses of the 3 tissues to Ang II are likely mediated by differential sex-based binding of Ang II to the angiotensin receptor type 1 (AT1R), a G protein–coupled receptor. As is true for most G protein–coupled receptors, the basal agonist state of their ligands modifies their response characteristics to additional stimulation, for example, agonist-mediated receptor downregulation (17). Thus, when the basal levels of a ligand are high, the receptor is desensitized, resulting in decreased responsiveness, and vice versa if its low (18). The mechanisms responsible for this change in receptor sensitivity could be secondary to changes in the receptor's secondary or tertiary structure, changes in the receptor density on the target cell surface, and/or recruitment of other factors that modify binding. Thus, for AT1R, if basal Ang II levels are low, then the response to acute changes in Ang II will be enhanced. The basal levels of PRA (and Ang II) levels are lower in women than men; therefore, as expected, the target tissues (glomerulosa cell, renal plasma flow, and BP) responses to Ang II (either infused or by standing up) were greater in women.

Additionally, recent studies have documented that intrinsic zona glomerulosa properties differ in men and women. First, the adrenal gland, and in particular the glomerulosa layer, is larger in women than men (19, 20). Second, ALDO's biosynthetic pathway in females differs from that of males. In contrast to the regulation of most steroids’ biosynthesis at the initial steps, for example, steroidogenic acute regulatory (StAR) protein and cholesterol side-chain cleavage enzyme (CYP11A1), ALDO's biosynthesis also is regulated at its last step, for example, ALDO synthase (CYP11B2). Females have higher expression levels both of CYP11A1 and StAR (20). Therefore, with periodic increases in Ang II due to posture, ACTH, etc, younger women and normotensive women can produce more ALDO than men.

### Why Are Basal Plasma Renin Activity (Angiotensin II) Levels Lower in Women Than in Men?

Based on the data summarized earlier, the lower PRA/Ang II in women vs men is likely secondary to volume expansion as indicated by women's increased SSBP. However, other factors could also be involved. For example, the relationship between renin and estrogen remains unclear. Although there are publications stating that estrogen stimulates the renin-angiotensin system by augmenting both tissue and circulating levels of angiotensinogen and renin (21), estrogen is also shown to counterregulate the classic, pressor angiotensin-converting enzyme/Ang II/AT1R pathway, which is upregulated in males, thereby playing a protective role against HTN and end-organ damage in women (22, 23). Contrariwise, the presence of estrogen does not explain the sex-related differences in PRA levels in the present study, as in older individuals, where estrogen levels are low, PRA levels were still lower in women than men.

### Are Women More Than Men at Risk for Being in a Relative Hyper-Aldosterone State?

The data available in HyperPATH are insufficient to definitively answer this question. Since men have higher ALDO level supine than women, if the individual's predominant daily status is supine, then, the men would have higher daily ALDO levels. Contrariwise, since the ALDO levels in women are higher in response to Ang II stimulation if the individual is upright and active, then women would have higher daily ALDO levels. For the entire data set, 24-hour urine ALDO levels were similar between men and women on a restricted-salt diet (*P* = .4) (see Table 1). These results would suggest that neither sex had a relative hyper-ALDO state on a daily basis. However, when stratified into subset groups, on a restricted-salt diet, normotensive women had greater 24-hour urine ALDO than normotensive men (*P* = .02), and women younger than 51 had higher 24-hour urine ALDO than men younger than 51 (*P* = .04) (see Supplementary Table S4) (12). These data suggest that it is a younger woman, theoretically in reproductive age, particularly if normotensive, who is more likely to be in a hyper-ALDO state. It is interesting to speculate if these results are related to the physiological needs of pregnancy. In a healthy pregnancy, plasma volume expansion and the increasing demands of a growing fetoplacental unit require an increase in renal blood flow (∼50% increase), increased sodium retention, and increased RAAS activation (see review (24)). However, although Ang II levels increase in pregnancy, ALDO levels increase disproportionally to PRA and thereby play a substantial role in the sodium retention required for the late stages of fetal growth (7).

### Clinical Implications

Women are more likely to have SSBP than men. However, women are NOT more likely to have increased daily ALDO production than men unless they are younger than 51. Therefore, if the physician identifies a woman younger than 51 with SSBP and HTN, likely the treatment of choice would be a mineralocorticoid receptor antagonist or potentially an ALDO synthase inhibitor (25).

### Limitations

Although there are major strengths of the HyperPATH cohort as has been discussed previously (8-10, 26), the most relevant limitation to our study is that it is observational and therefore hypothesis-generating not -testing. Even though several other observational studies have also reported that women have more SSBP than men, confirmation of the proposed mechanism(s) reported herein requires a prospective study. The present study assumes that estrogen and progesterone have limited, if any, involvement in the proposed ALDO mechanism since in those aged 51 years or older ALDO levels were still higher basally in men than in women, but higher in women following Ang II stimulation. Again, a meticulously designed prospective study would be required to provide more substantial evidence.

### Summary

Women had greater SSBP, greater ALDO response to Ang II, and higher ALDO/PRA ratios than men, but men had higher levels of basal ALDO and its secretagogues (PRA/Ang II, potassium, and cortisol/ACTH). Twenty-four–hour urine ALDO levels were used to assess overall ALDO production. They were the same in men and women in the total population. However, when stratified into subset groups, normotensive women vs men had greater urine ALDO levels, and women vs men younger than 51 had higher urine ALDO levels. These results suggest increased ALDO production is the mechanism responsible for the SSBP in women but only in those younger than 51. A prospective clinical study and an appropriately designed clinical trial would be required to document this hypothesis further. In older individuals the mechanisms underlying the increased SSBP in women vs men are uncertain.

## Data Availability

Values for all data points for each graph are shared in the data repository (27).

## Abbreviations

ACTH: adrenocorticotropic hormone
ALDO: aldosterone
Ang II: angiotensin II
ARR: aldosterone/plasma renin activity
AT1R: angiotensin receptor type 1
BMI: body mass index
HTN: hypertension
K^+^: potassium
PRA: plasma renin activity
RAAS: renin-angiotensin-aldosterone system
SBP: systolic blood pressure
SSBP: salt-sensitive blood pressure
